# Structure and expression pattern of *Oct4 *gene are conserved in vole *Microtus rossiaemeridionalis*

**DOI:** 10.1186/1471-2164-9-162

**Published:** 2008-04-11

**Authors:** Sergey P Medvedev, Alexander I Shevchenko, Eugene A Elisaphenko, Tatyana B Nesterova, Neil Brockdorff, Suren M Zakian

**Affiliations:** 1 SD Russian Academy of Sciences, Institute of Cytology and Genetics, ac. Lavrentiev ave.10, 630090 Novosibirsk, Russia; 2 MRC Clinical Sciences Centre, ICFM Hammersmith Hospital, Du Cane Road, W12 0NN, London, UK

## Abstract

**Background:**

Oct4 is a POU-domain transcriptional factor which is essential for maintaining pluripotency in several mammalian species. The mouse, human, and bovine *Oct4 *orthologs display a high conservation of nucleotide sequence and genomic organization.

**Results:**

Here we report an isolation of a common vole (*Microtus rossiaemeridionalis) Oct4 *ortholog. Organization and exon-intron structure of vole *Oct4 *gene are similar to the gene organization in other mammalian species. It consists of five exons and a regulatory region including the minimal promoter, proximal and distal enhancers. Promoter and regulatory regions of the vole *Oct4 *gene also display a high similarity to the corresponding regions of *Oct4 *in other mammalian species, and are active during the transient transfection within luciferase reporter constructs into mouse P19 embryonic carcinoma cells and TG-2a embryonic stem cells. The vole *Oct4 *gene expression is detectable starting from the morula stage and until day 17 of embryonic development.

**Conclusion:**

Genomic organization of this gene and its intron-exon structure in vole are identical to those in all previously studied species: it comprises five exons and the regulatory region containing several conserved elements. The activity of the *Oct4 *gene in vole, as well as in mouse, is confined only to pluripotent cells.

## Background

The transcription factor Oct4, known also as Oct3 and Oct3/4, belongs to class V of the POU (Pit, Oct, Unc) transcription factor family. The POU family includes transcription factors containing the POU domain and regulating transcription via binding to an octamer motif located in the promoter or enhancer regions of target genes [1,2].

An important role of Oct4 factor in sustaining pluripotency of preimplantation embryonic cells and mouse embryonic stem cells (ES cells) has been convincingly demonstrated using the directed mutagenesis and RNA interference [3-5]. Embryos homozygous for *Oct4 *gene mutation die during implantation because of the inability to form the inner cell mass (ICM) of the blastocyst [3]. Suppression of *Oct4 *expression in ES cells by RNA interference induces cell differentiation into trophectodermal derivatives [4].

The mouse, human, and bovine *Oct4 *orthologs have a highly conserved nucleotide sequence and genomic organization [6-8].

*Oct4 *gene expression in preimplantation mouse embryo is confined exclusively to pluripotent ICM cells [9]. After the implantation, *Oct4 *expression in somatic tissues reduces and remains only in primordial germ cells. In addition, expression of *Oct4 *is characteristic of embryonic stem (ES) cell lines, embryonic carcinoma (EC) cells, and embryonic germ (EG) cell lines [2,10]. Unlike mouse, the *Oct4 *expression in human, cattle, and swine preimplantation embryos is not restricted to ICM, but is also observed in trophectoderm cells [11-13].

*Oct4 *expression is regulated at the transcriptional level by *cis*-regulatory elements located in the 5' region of the gene [14-16]. Analysis of *LacZ *reporter gene expression, controlled by a fragment of the mouse *Oct4 *genomic locus, revealed two elements essential for regulating the cell-specific expression of *Oct4 *gene. These elements were named as proximal and distal enhancers. The studies have shown that distal enhancer is active in ICM, ES, EG and primordial germ cells, whereas the proximal enhancer is needed to activate *Oct4 *gene in embryonic ectoderm and mouse EC cells [15]. So far the structure, expression and regulation of the *Oct4 *gene have been studied comprehensively in mouse, human, and cattle [7,17,18]. In this work, a genomic copy and cDNA of the vole *Oct4 *gene were isolated and cloned as well as its exon-intron structure and expression were studied. Nucleotide sequences of the coding and regulatory regions of vole *Oct4 *gene were compared to the corresponding sequences of six mammalian species, including mouse, rat, human, chimpanzee, cattle, and dog. Use of luciferase reporter constructs allowed demonstrated that individual elements of the regulatory region of vole *Oct4 *gene were functionally active in mouse pluripotent cells. This fact suggests a high conservation of the system regulating this gene in mammals. A comprehensive study of the genes involved in maintaining pluripotency such as *Oct4 *in voles will make it possible to obtain new information about the species-specific features of their structure, expression, and regulation, and thereby enhancing optimization of the experiments on obtaining of vole ES cells.

## Results

### Nucleotide sequence, exon-intron structure, and expression of *M. rossiaemeridionalis Oct4 *gene

**Figure 1:**
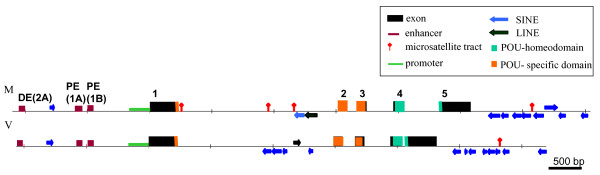
***Oct4 *****genes organization in mouse (M) and vole (V).**

Clone containing genomic sequence of vole *Oct4 *was isolated via screening of the *M. rossiaemeridionalis *genomic phage library. Overall, 10315 bp were identified, where five exons and the regulatory region of *Oct4 *gene were conditionally mapped based on the homology to mouse *Oct4 *gene sequence. The coding region contains no stop codons, and canonical splice sites are present at the putative exon-intron boundaries. The minimal promoter, as well as proximal and distal enhancers were identified within the regulatory region on the basis of comparative sequence analysis. Repeated DNA sequences were found in introns and in the 5' and 3' regions. These repeats are represented mainly by SINE elements (Figure 1). The sequences located 3' to exon 5 of vole *Oct4 *gene, which were sequenced only partially, displayed a similarity to the major histocompatibility complex.

To verify the exon-intron structure of vole *Oct4 *gene, 3'- and 5'-RACE experiments were performed. Preimplantation embryos of *M. rossiaemeridionalis *were used as a source of RNA. Comparison of cDNA clones obtained by RACE with the genomic sequence confirmed that vole *Oct4 *gene contained five exons corresponding to the *Oct4 *exons of other mammalian species studied (Figure 1). The splicing sites of vole *Oct4 *gene transcript are conserved and follow the GT/AG rule. The detected transcript is 1357 nt long and encodes for a protein with a length of 354 aa. The 5' UTR is 71 bp long; 3' UTR, 224 bp. Relative to mouse, the transcription start site of vole *Oct4 *is located 23 bp farther upstream falling within an 8-bp insertion, specific to the vole minimal promoter. The polyadenylation signal of vole *Oct4 *has a species-specific sequence; the 3' boundary of the gene is located 7 bp upstream of the mouse gene. In all investigated samples, 2-cell embryos, morulas, blastocysts, primordial gonads of postimplantation embryos on day 8–17dpc, adult organs (liver, kidney, spleen, intestine and testicles), cultures of R1 trophoblast stem cells and extraembryonic endoderm cells no products of alternative splicing were found for vole *Oct4 *gene.

**Figure 2:**
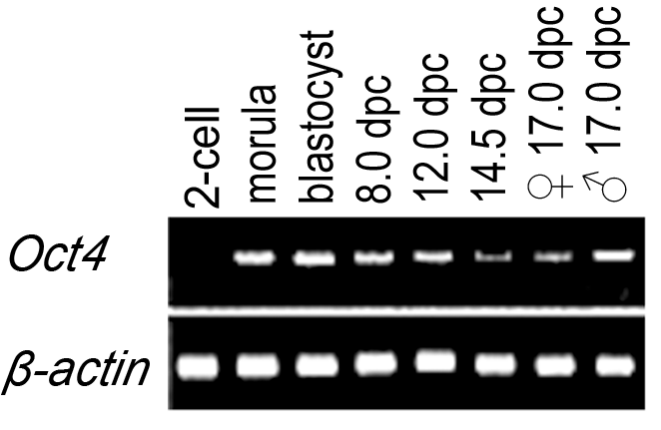
**Analysis of the *Oct4 *gene expression in the embryogenesis of vole *M. rossiaemeridionalis *by 3'-RACE method**. 3'-RACE products: RNA sources for the experiment: vole embryos at the stage of 2 cells; morulas; blastocysts; 8.0 dpc embryos; 12.0 dpc; 14.5 dpc; 17.0 dpc ovary; 17.0 dpc testis.

3'-RACE allowed us to detect the expression of vole *Oct4 *gene during preimplantation development at the morula and blastocyst stages (2.5–4.5 dpc) and in genital ridges of postimplantation stages from 8 dpc up to 17 dpc (Figure 2). Transcription of *Oct4 *gene was undetectable in the organs of adult voles (the liver, kidneys, spleen, intestines, and testicles), trophoblast stem cells, and extraembryonic endoderm cells (data not shown).

### Analysis of the activity of vole *Oct4 *promoter

**Figure 3:**
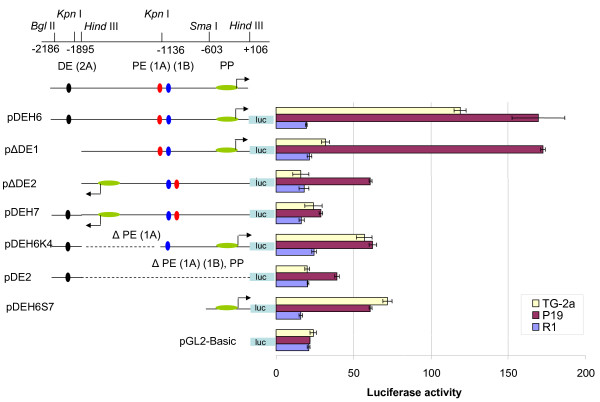
**Analysis of the activity of luciferase reporter constructs carrying different elements of the regulatory region of *Oct4 *gene in TG-2a, P19 and R1 cell lines**. PP, proximal promoter; PE (1A) (1B), proximal enhancer, sites 1A and 1B; and DE (2A), distal enhancer, site 2A.

To analyse the role of putative regulatory elements of vole *Oct4 *gene, we have used a luciferase reporter assay (Figure 3). Pluripotent ES cell lines have not been derived for vole species, therefore we transiently transfected luciferase reporter vectors containing various elements of vole *Oct4 *putative regulatory region into mouse ES cell line TG-2a, mouse EC cells P19, and vole trophoblast stem cells R1. An empty vector pGL2-Basic has been used as a negative control. In pluripotent mouse TG-2a cells, pDEH6 plasmid, containing promoter and both enhancers (-2186/+106) in a direct orientation with respect to luciferase gene, displayed the highest luciferase activity, which exceeded the background level (compare to luciferase activity of empty vector pGL2-Basic) approximately six-fold (Figure 3). Similar, if not even more pronounced effect was observed when the same construct was transfected into mouse EC cells. Deletion of the distal enhancer (p DE1, -1895/+106) almost completely abolishes the promoter activity in TG-2a cells, however it does not have any effect on the activity in P19 EC cells that demonstrate equally high luciferase activity as a complete construct. This observation is in line with the data obtained for mouse which indicate the crucial role of distal enhancer for *Oct4 *activity in ES cells [15].

Deletion of proximal enhancer (PE 1A, pDEH6K4) or both distal and proximal ehancers (pDEH6S7) reduce the promoter activity 2–3 folds, demonstrating that the vole *Oct4 *promoter is capable of promoter activity in pluripotent cells by itself, but at significantly lower level (Figure 3). The lowest activity comparable to the background was observed in the case of constructs pDEH7 and pDE2. The promoter and proximal enhancer in clone pDEH7 were in the opposite orientation to the gene, and this region (-1895/+106) was deleted in clone pDE2. Consistent with the lack of *Oct4 *expression in trophoblast cells, no luciferase activity was detected when constructs were transfected into vole TS cell line R1. The data obtained in this experiment indicate that, similar to mouse, vole Oct4 regulatory elements are necessary for efficient and cell type specific expression of the gene.

### Comparative sequence analysis

**Figure 4:**
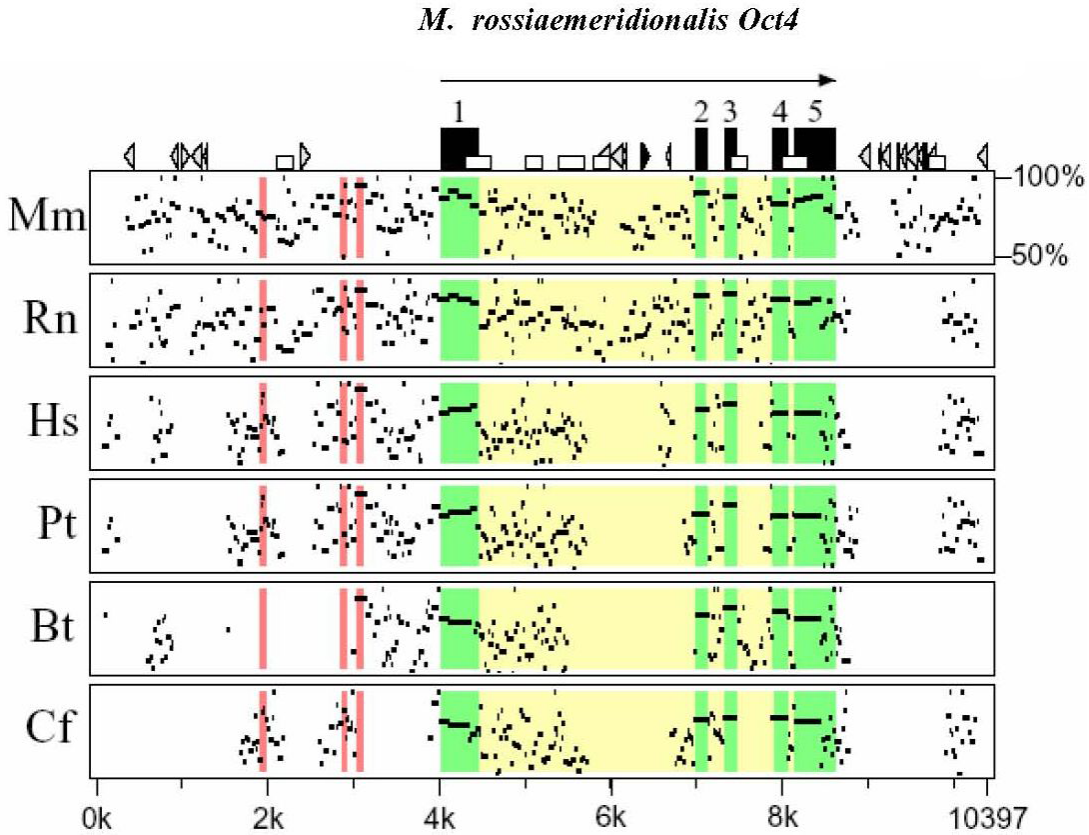
**The percent identity plot of the *Oct4 *gene sequences in different mammalian species**. Designations: →, the direction of gene transcription; ▬, exons; ▭, CpG islands; ►, dispersed MIR repeats; ▻, other SINE elements; and , long terminal repeats. Regions of the regulatory elements of vole *Oct4* gene are shown in red. Black rectangles denote the exons of vole *Oct4* gene; arrow indicates the direction of gene transcription. Various geometric figures (triangles, rectangles) stand for different genetic elements composing the nucleotide sequence. The linear nucleotide sequence of *Oct4* gene for each species is plotted on the abscissa axis. Arrangement of dots on the ordinate reflects the homology level between the *Oct4* gene sequence of a species and the vole sequence in the range of 50 to 100% (the higher is the dot position, the higher is the homology).**Mr**, *M. rossiaemeridionalis*; **Mm**, *Mus musculus*; **Rn**, *Rattus norvegicus*; **Hs**, *Homo sapiens*; **Pt**, *Pan troglodytes*; **Bt**, *Bos taurus*; and **Cf**, *Canis familiaris*.

Vole *Oct4 *nucleotide sequence was aligned with *Oct4 *sequences of mouse, rat, human, chimpanzee, cattle, and dog, obtained from GenBank (Figure 4 and Table 1). Overall nucleotide sequence homology varies between 81% (vole v. dog) and 89% (vole v. rat), however the homology is much higher for the exons 2 and 3, encoding for DNA-binding POU domain in all pairs of species compared (Table 1). The homology is lower (55–70%) in intronic as well as 5' and 3' UTR regions, especially where species-specific mobile elements are localized (Figure 4).

**Table 1:** The homology (%) between the nucleotide/amino acid sequences of the *Oct4 *coding region of vole and other six mammalian species

Pairs of species compared	*Oct4 *exons					Mean homology	Homology in POU-domain
	1	**2**	**3**	4	5		

*Mr/Mm*	83/80	**95/95**	**91/97**	86/81	79/85	87/80	90/91
*Mr/Rn*	84/82	**95/95**	**92/97**	88/84	84/87	89/87	91/93
*Mr/Hs*	80/80	**87/92**	**87/97**	83/90	71/80	83/85	87/94
*Mr/Pt*	79/78	**86/90**	**87/95**	79/86	83/78	82/83	86/92
*Mr/Bt*	75/72	**85/90**	**88/97**	87/94	70/78	81/82	88/94
*Mr/Cf*	75/70	**85/90**	**87/97**	88/92	81/77	81/80	88/94

***Mr***, *M. rossiaemeridionalis*; ***Mm***, *Mus musculus*; ***Rn***, *Rattus norvegicus*; ***Hs***, *Homo sapiens*; ***Pt***, *Pan troglodytes*; ***Bt***, *Bos taurus*, and ***Cf***, *Canis familiaris*

Comparison of the Oct4 amino acid sequences demonstrates relatively high similarity level for the POU-specific domain for all species under study (encoded by exons 2 and 3, see Figure 1 and Table 1). Surprisingly, the highest homology is observed between vole and cattle (95%) and the lowest, between vole and mouse (91%). Thus, the homology between the amino acid sequences of POU domain from evolutionarily more distant species (vole-cattle, vole-human, vole-chimpanzee, and vole-dog) appeared higher as compared to the pairs of evolutionarily related species belonging to the same order (vole-mouse, vole-rat). When comparing the total homology of Oct4 proteins, we found that vole and rat are the most similar (87%). It is of considerable interest that mouse Oct4 protein demonstrates the least amino acid homology with closely related vole (81%), as well as with all other species studied (data not shown). Such a low similarity between mouse and vole Oct4 proteins contrasts with much higher nucleotide homology (87%) between *Oct4 *genes in these species.

**Figure 5:**
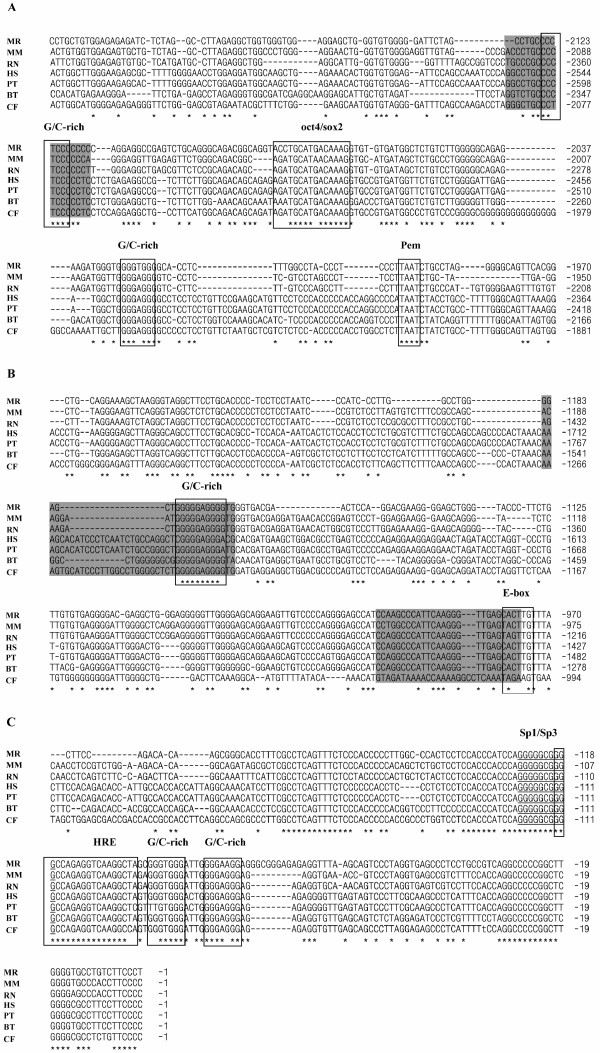
**Alignments of nucleotide sequences of the regulatory regions the *Oct4 *genes belonging to seven mammalian species**. **Mr**, *M. rossiaemeridionalis*; **Mm**, *Mus musculus*; **Rn**, *Rattus norvegicus*; **Hs**, *Homo sapiens*; **Pt**, *Pan troglodytes*; **Bt**, *Bos taurus*; and **Cf**, *Canis familiaris*. A, distal enhancer, site 2A, autoregulatory OCT4/SOX2-element; B, proximal enhancer, sites 1A and 1B; C, the region of minimal promoter.

Next we compared the regions important for *Oct4 *gene regulation and function (Figure 5). Previously it was demonstrated that three motifs (GGGAGGG in the proximal enhancer, CCCTCCC the distal enhancer, and GGGGGCGGGG in the minimal promoter) represent transcription factor binding sites in mouse ES and EC cells [19]. Treatment of undifferentiated ES and EC cells with retinoic acid causes loss of transcription factor binding to these motifs, thereby suppressing *Oct4 *gene expression and leading to cell differentiation. Therefore we have analysed the conservation of these three motifs as well as several other motifs located in the regulatory region.

The homology between the minimal promoters varies between 83–98% in different mammalian species. In vole and other mammals, the GGGGGCGGGG motif (positions -126/-117 in vole) within the minimal promoter is the most conserved (Fig. 5C). It is a potential transcription factor binding site for the factors of Sp family, partially overlaps the hormone responsive element (HRE) (positions -119/-101 in vole), is sensitive to retinoic acid, and is recognized by some nuclear receptors [8,14,15,19,20] (Fig. 5C). However, not all the elements of *Oct4 *gene basal promoter exhibit similar conservation. The GGGTGGG motif (-98/-92) is 100% homologous in all the species except for dog. The CCCACCC motif (-159/-153) is identical in mouse, cattle, and dog; but it is different in rat, human, and chimpanzee. The GGGAGGG site (positions -76/-71 in the mouse sequence) identical in the six species compared but is partially changed in vole, being represented by GGGAAGG sequence. At a distance of one nucleotide from this site in the vole sequence, another GC-rich site is located, GGGCGGG (-79/-73); this site is absent in the sequences of other species (Figure 5C).

Upstream the basal promoter, the two motifs display a high conservation, namely, CCCTCCC (-2125/-2119), which is identical in all species except for dog, and GGGAGGG (-1176/-1170). These motifs are located in the distal and proximal enhancers, respectively. In addition, several other GC-rich motifs of CCC(A/T)CCC and GGG(A/T)GGG types with different levels of homology to the sequences of other species were detected in vole. Two overlapping GGGAGGGGTGGG sites are located at positions -1176/-1165 of vole *Oct4 *gene regulatory region; the site GGGAGGG (-1176/-1170) from this region displays a 100% homology in all the species compared. As for the site GGGTGGG (-1171/-1165), it is completely homologous only in vole, rat, and mouse. Superposed GGGTGGGGTGGG sites are also located at positions -2031/-2020. Both parts of this superposed site are changed in other species; however, in the site with positions -2026/-2020, all the species except for vole contain the substitution of thymine for with adenine. This results on formation of GGGAGGG site, which is also potentially functional (Figure 5A, B and Table 2).

**Table 2:** The homology (%) between nucleotide sequences of the elements in regulatory regions of *Oct4 *genes of seven mammalian species

		***Cf***	***Hs***	***Mm***	***Mr***	***Pt***	***Rn***
***Bt***	PP	90	88	88	86	86	86
	PE1A	77	67	75	78	77	75
	PE1B	-	100	92	96	100	92
	DE2A	81	85	74	88	85	84
	OCT4/SOX2	93	93	87	87	93	93
***Cf***	PP		88	86	86	86	84
	PE1A		64	81	89	71	81
	PE1B		-	-	-	-	-
	DE2A		81	68	81	81	74
	OCT4/SOX2		100	93	93	100	100
***Hs***	PP			85	85	98	85
	PE1A			67	68	93	67
	PE1B			92	96	100	92
	DE2A			84	94	100	84
	OCT4/SOX2			93	93	100	100
***Mm***	PP				88	83	96
	PE1A				84	76	86
	PE1B				88	92	92
	DE2A				94	84	89
	OCT4/SOX2				87	93	93
***Mr***	PP					83	87
	PE1A					79	89
	PE1B					96	88
	DE2A					94	94
	OCT4/SOX2					93	93
***Pt***	PP						83
	PE1A						76
	PE1B						92
	DE2A						84
	OCT4/SOX2						100

***Mr***, *M. rossiaemeridionalis*; ***Mm***, *Mus musculus*; ***Rn***, *Rattus norvegicus*; ***Hs***, *Homo sapiens*; ***Pt***, *Pan troglodytes*; ***Bt***, *Bos Taurus*, and ***Cf***, *Canis familiaris*; and (-) no data

Five so-called E boxes, having a 5'-CANNTG-3' consensus and being potential binding sites for transcription factor Mash-2 [21] were detected in the 5' region of vole *Oct4 *gene (-2547/-1). The most conserved E box is located at positions -332/-327 and -978/-973 of the vole *Oct4 *gene regulatory region; it retains the consensus in the four species compared (no data are available for dog). The E box at positions -978/-973 is identical to human, chimpanzee, and bovine sequences, but differs in mouse and rat. The E box at positions -479/-474 displays a 100% homology to human and chimpanzee sequences and is absent in mouse and rat sequences. Potential binding sites for transcription factor Pem, with an ATTA consensus, were detected in the regulatory region of vole *Oct4 *gene at positions -665/-662, -1899/-1894, -1995/-1992, -2254/-2251, -2373/-2370, and -2541/-2538. The ATTA site located at positions -1995/-1992 displays an absolute homology in all species; the site at positions -2254/-2251 shows a 100% homology to human, chimpanzee and bovine sequences; and at positions -2373/-2370 is identical to the mouse sequence.

In the 5' region of vole *Oct4 *gene, an autoregulatory composite site was detected, containing potential binding sites for transcription factors Oct4 (5'-AGATGCAT-3') and Sox2 (5'-GACAAAG-3'). Electrophoretic mobility shift assays and chromatin immunoprecipitation experiments indicate that transcription factors Oct4 and Sox2 bind directly to the composite Oct4/Sox2 elements in *Oct4 *and *Sox2 *genes in mouse and human ES cells [22,23]. In vole, this site is located at positions -2081/-2067 and displays a 87–93% homology to the sequences found in regulatory regions of other mammalian species (Figure 5A and Table 2).

The sequences located upstream of the *Oct4 *minimal promoter are not conserved; their homology reflects the degree of evolutionary relationship of the species compared. Nevertheless, the short sequences corresponding to site 2A of the distal enhancer and sites 1A and 1B of the proximal enhancer are distinctly detectable even in the evolutionally distant species. The homology between vole and the species taken for comparison varies from 81 to 94% in the region of site 2A of the distal enhancer; in the region of proximal enhancer site 1A, from 68 to 89%; and in the region of proximal enhancer site 1B, from 88 to 96% (Table 2).

## Discussion

In this work, an ortholog of the vole *M. rossiaemeridionalis *gene encoding transcription factor *Oct4*, which is involved in sustaining the pluripotent cell state at the early stages of embryogenesis, has been isolated and studied. Genomic organization of this gene and its intron-exon structure in vole are identical to those in all previously studied species: it comprises five exons and the regulatory region containing several conserved elements. A tight coupling with the major histocompatibility complex is typical of the vole *Oct4*, as well as of its orthologs in mouse, cattle, and human [7,17,18].

Comparative analysis of nucleotide sequences and the experiments with transient transfection of *Oct4 *gene promoter and its regulatory regions within reporter constructs demonstrate once again the conservation of these sequences and their functions. Individual substitutions and insertions in this region detected in the vole *Oct4 *do not prevent transcription of this gene in the pluripotent mouse cells. The element that is absolutely necessary for the activity of this gene is the region of minimal promoter (-527/-1); the other elements cannot provide the transcription by themselves and are only able to modulate its level. Moreover, the activation and modulation of transcription depend not only on the presence of certain elements, but also on their correct orientation relative to the transcription start site. In addition, the results obtained demonstrate that *Oct4 *regulatory regions function in different types of pluripotent cell. A lower activity of the construct with deleted distal enhancer in TG-2a cells versus P19 line cells supports the idea that the distal enhancer is a key element providing a high activity of *Oct4 *gene in embryonic stem cells [15].

The comparative analysis of the nucleotide sequences of *Oct4 *orthologs in seven mammalian species demonstrated a high conservation of the genomic organization, coding region, and the main elements (minimal promoter, distal, and proximal enhancers) of *Oct4 *gene regulatory region. Phylogenetic sequence analysis of the promoter regions showed that the most conserved elements in this region are the sites for transcription factors of Sp1 family and the hormone-responsive element, which retain high similarity and similar organisartion. In addition, a high homology of GC-rich motifs and autoregulatory Oct4/Sox2 site was observed, whereas the homology of other elements in the regulatory region, in particular, the sites for transcription factors Mash-2 and Pem, is rather limited. This fact may suggest that the GC-rich motifs and autoregulatory Oct4/Sox2 site are the elements that are most essential for the regulation of *Oct4 *gene expression.

Note that *Oct4 *is the most conserved among the genes involved in sustaining the pluripotency. For comparison, the *Nanog *gene of mouse and human shows a 58% similarity on their nucleotide sequence in the gene coding region [24]. However, despite the conservation of these sequences, considerable interspecific differences in expression of this gene are detectable in ontogenesis.

Expression of this gene in vole is detected at the early preimplantation (morula, blastocyst) stages and also in the genital ridges of postimplantation embryos. Presumably, the expression of *Oct4 *gene in postimplantation vole embryos depends on its activity in primordial germ cells, the gamete precursors, as was shown also for mouse [25]. Expression of vole *Oct4 *gene is undetectable in the organs of adult animals and somatic cell lines, unlike the human *Oct4*, which is detected in adult tissues [17,18]. The vole *Oct4 *transcript shows no alternative variants of splicing, which is characteristic of human [18]. Since the expression of this gene was not found in the trophoblast stem cell lines and extraembryonic endoderm lines of *M. rossiaemeridionalis*, this suggests that, at the stage of late blastocyst, this gene in vole, as well as in mouse, is expressed only in epiblast cells. Conceivably, the epiblast cells in vole late blastocysts retain the pluripotent state at least up to the start of implantation, which allows expecting a successful obtaining of ES cells in this species.

## Conclusion

In this study, we cloned, sequenced and an alysed vole ortholog of *Oct4 *gene. A special attention was focused on studying the regulatory region of *Oct4 *gene using reporter constructs and comparative sequence analysis. The comparison involved a large number of species (seven, including the vole sequence that we determined) belonging to various families, which allowed us to detect the most evolutionarily conserved elements in the regulatory region containing the potential transcription factor binding sites. Using the reporter constructs, we have demonstrated that the enchanter element and promoter of vole *Oct4 *gene are active in mouse pluripotent cells. We analysed vole *Oct4 *gene expression in early preimplantation embryos, genital ridges of postimplantation embryos, adult tissues and stem cell lines precursors of trophoblast and extraembryonic endoderm. We have found expression only in preimplantation embryos and in genital ridges, containing primordial germ cells, consistent with *Oct4 *being expressed specifically in pluripotent cells.

## Methods

### *Microtus rossiaemeridionalis *genomic library screening

Lambda DASH II genomic library of vole *M. rossiaemeridionalis *[26] has been screened according to a conventional method [27] using vole genomic PCR fragment as a probe. The PCR fragment has been amplified with the primers Oct2F (5'-ccaagctgctgaagcagaaga-3') and Oct5R (5'-tttgaatgcatgggagagccca-3') designed from the most conserved region of mouse and human *Oct4 *exons 2 and 5. Sequence analysis revealed that amplified 631 bp vole fragment is a processed *Oct4 *pseudogene, since its sequence shows homology to the mouse *Oct4 *transcript, does not contain introns and includes several stop codons. The screening gave several clones from which the clone containing full *Oct4 *gene with surrounding sequences was selected by PCR analysis. The nucleotide sequence of the clone was determined according to the protocol *ABI PRISM BigDye™ Terminator Cycle Sequencing Ready Reaction Kit *(Applied Biosystems, Perkin-Elmer Corporation) in an *ABI PRISM 377 *automated sequencer using universal and gene-specific primers. Sequence data has been processed using SeqMen program (DNASTAR Inc.). The clones, carrying Oct4 pseudogenes were not used in the further study. The genomic and cDNA sequences of *M. rossiaemeridionalis *gene *Oct4 *is deposited with the GeneBank under accession numbers: EF032593 and EF030115, respectively.

### Cell cultures and media

TG-2a mouse ES cells were cultured in GMEM (Invitrogen) supplemented with 15% fetal calf serum (FCS; Autogen Bioclear, UK), 1% non-essential amino acids, 1 mM L-glutamine, 50 U/ml penicillin-streptomycin, 0.1 mM 2-mercaptoethanol, and 10^3 ^U/ml ESGRO (Chemicon). P19 cells were grown in MEM (Invitrogen) supplemented with 10% FCS and 50 U/ml penicillin-streptomycin. Vole trophoblast stem cells (R1) were cultivated in the medium containing 40% DMEM (Invitrogen), 40% F12 medium, 15% FCS, 1% nonessential amino acids, 50 U/ml penicillin-streptomycin, 1 mM L-glutamine, nucleotides (0.03 mM ATP, 0.01 mM TTP, 0.01 mM GTP, and 0.01 mM CTP), and 1000 U/ml vole leukemia inhibitory factor (LIF). The cells were grown in gelatinised flasks.

### Reporter vector construction

To obtain a reporter construct containing distal enhancer of the vole *Oct4 *gene (pDE2) fragment of regulatory region (-2186/-1895) was cloned into pGL2-Basic vector (Promega) in *Bgl*II-*Hind*III sites at the 5' region of luciferase gene. To produce a construct enclosing all elements of the regulatory region a (-1895/+106) fragment, containing proximal enhancer and promoter region of vole *Oct4 *gene, was cloned into plasmid pDE2 at *Hind*III site (pDEH6 construct). The (-1895/+106) fragment was also cloned into pDE2 in an opposite orientation (pDEH7 construct). In addition, a fragment (-1895/+106) alone was cloned into pGL2-Basic vector in both orientations relative to luciferase gene (constructs pΔDE1 and pΔDE2, respectively). Deletion of 1A site in proximal enhancer was achieved by *Kpn*I digestion and following self-ligation of pDEH6 plasmid, producing pDEH6K4. pDEH6S7 plasmid, containing only the promoter region (-603/+106) of vole *Oct4 *gene, was obtained by excision of *Sma*I fragment from pDEH6 plasmid.

### Transient transfection with reporter vectors

TG-2a mouse ES cells, P19 mouse EC cells, and R1 vole trophoblast stem cells were plated into six-well plates (Nunc) at 0.5 × 10^6 ^cells per well and cultivated for 24 hrs. Each reporter construct (4 μg/well) was transfected into cells using Lipofectamin2000 (Invitrogen). Cell lysate was prepared 48 hrs after the transfection; luciferase activity was detected using a Luciferase Assay System (Promega). Transfection of the pGL2-Basic vector (Promega) without insertion was used as a control of the basic level of luciferase activity. Each transfection experiment was performed in triplicate.

### RNA isolation, RT-PCR, 3'- and 5'-RACE

Vole preimplantation embryos were collected in 50 μl of sterile PBS (1.7 mM KH_2_PO_4_, 5.5 mM Na_2_HPO_4_, and 150 mM NaCl). RNA was isolated from morula, blastocysts, and primordial gonads of postimplantation embryos on day 8–17dpc, from adult organs (liver, kidney, spleen, intestine and testicles), cultures of R1 trophoblast stem cells and extraembryonic endoderm cells using TRI-REAGENT (Sigma). RNA samples were treated with DNAse to eliminate DNA contamination (TurboDNA-free kit, Ambion). cDNA was synthesized using SuperScript III reverse transcriptase (Invitrogen). 5'- and 3'-RACE were performed with BD SMART™ RACE cDNA Amplification Kit (Clontech). Gene-specific primers used for *Oct4 *RACE were oct4_3'RACE1: (5'-tggagaagtgggtggaggaagccgacaaa-3') and oct4_5'RACE2 (5'-ggttgggggtcacgccgttctcaat-3'); strandspecific primer for *β-actin *cDNA synthesis was: Bass (5'-acacgcagctcattgtac-3'), PCR primers for *β-actin *were: BA11 (5'-gatatcgctgcgctggtcgt-3') and BA2 (5'-agatcttctccatgtcgtcc-3') [28]. Vole genomic DNA was used as RACE negative control. The RACE products were cloned into pCR4-TOPO vector (Invitrogen). The nucleotide sequence of clones was determined in both directions with universal primers.

### Comparative genomics

Vole *Oct4 *locus was analyzed for the presence of repeated sequences using the RepeatMasker program [29]. The genome *Oct4 *sequences of mouse, rat, human, chimpanzee, cattle, and dog, were obtained from UCSC Genome Bioinformatics Site [30]: mouse mm8_dna assembly chr17:35109361–35123778; rat rn3_dna assembly chr20_random:1–12233; human hg17_dna assembly chr6:31235110–31251418; chimpanzee panTro1_dna assembly chr5:31700540–31716543; cattle Tau2_dna assembly chr23:20473820–20488846; dog Fam2_dna assembly chr12:3859822–3874546, and used in comparative analysis. The *Oct4 *sequences of different species were analyzed with Pip Maker program [31]. CLUSTALX was used for constructing multiple alignments of the sequences. Pairwise comparison of nucleotide and amino acid sequences was carried out with FASTA [32].

## List of abbreviations used

UTR, untranslated regions; PCR, polymerase chain reaction; GMEM, Glasgow minimum essential medium; MEM, minimum essential medium; FCS, fetal calf serum; PBS, phosphate-buffered saline; RACE, rapid amplification of cDNA ends; RT-PCR, reverse-transcriptase polymerase chain reaction;
